# Impact of Computer-Assisted System on the Learning Curve and Quality in Esophagogastroduodenoscopy: Randomized Controlled Trial

**DOI:** 10.3389/fmed.2021.781256

**Published:** 2021-12-14

**Authors:** Li Huang, Jun Liu, Lianlian Wu, Ming Xu, Liwen Yao, Lihui Zhang, Renduo Shang, Mengjiao Zhang, Qiutang Xiong, Dawei Wang, Zehua Dong, Youming Xu, Jia Li, Yijie Zhu, Dexin Gong, Huiling Wu, Honggang Yu

**Affiliations:** ^1^Department of Gastroenterology, Renmin Hospital of Wuhan University, Wuhan, China; ^2^Key Laboratory of Hubei Province for Digestive System Disease, Renmin Hospital of Wuhan University, Wuhan, China; ^3^Hubei Provincial Clinical Research Center for Digestive Disease Minimally Invasive Incision, Renmin Hospital of Wuhan University, Wuhan, China; ^4^Nursing Department of Renmin Hospital of Wuhan University, Wuhan, China; ^5^Department of Gastroenterology, Xiaogan Chinese Medical Hospital, Xiaogan, China

**Keywords:** endoscopy, training, artificial intelligence, learning curve, esophagogastroduodenoscopy

## Abstract

**Background and Aims:** To investigate the impact of the computer-assisted system on esophagogastroduodenoscopy (EGD) training for novice trainees in a prospective randomized controlled trial.

**Methods:** We have constructed a computer-aided system (CAD) using retrospective images based on deep learning which could automatically monitor the 26 anatomical landmarks of the upper digestive tract and document standard photos. Six novice trainees were allocated and grouped into the CAD group and control group. Each of them took the training course, pre and post-test, and EGD examination scored by two experts. The CAD group was trained with the assistance of the CAD system and the control group without.

**Results:** Both groups achieved great improvements in EGD skills. The CAD group received a higher examination grading score in the EGD examination (72.83 ± 16.12 vs. 67.26 ± 15.64, *p* = 0.039), especially in the mucosa observation (26.40 ± 6.13 vs. 24.11 ± 6.21, *p* = 0.020) and quality of collected images (7.29 ± 1.09 vs. 6.70 ± 1.05). The CAD showed a lower blind spot rate (2.19 ± 2.28 vs. 3.92 ± 3.30, *p* = 0.008) compared with the control group.

**Conclusion:** The artificial intelligence assistant system displayed assistant capacity on standard EGD training, and assisted trainees in achieving a learning curve with high operation quality, which has great potential for application.

**Clinical Trial Registration:** This trial is registered at https:/clinicaltrials.gov/, number NCT04682821.

## Background

Hundreds of millions of esophagogastroduodenoscopy (EGD) procedures are performed every year worldwide and play a pivotal role in the diagnosis and management of upper gastrointestinal disorders (1). The effectiveness of EGD depends on the endoscopists' skills, which need a prolonged learning curve for novice trainees to perform high-quality endoscopy care. Despite the technical skills and non-technical skills, such as communication and teamwork needed for endoscopy manipulation, EGD training also requires quality control and cognitive skills, such as low blind spot rate, which is a significant indicator for EGD quality (2, 3).

Previous studies have focused on the successful use of endoscopic simulators for training EGD in the past decades, including *ex vivo* animal tissue models, live animal models, mechanical models, and virtual reality computer simulators (3–10). Even though the value of validated simulators has been proven in pre-patient EGD training, patient-based training procedures are necessary (11). To date, a real-time aided training system in patient-based EGD is still lacking.

Deep learning, which is essentially a neural network, attempts to simulate the behavior of the human brain, albeit far from matching its ability, allowing it to “learn” from large amounts of data. Deep learning drives many artificial intelligence (AI) applications to conduct analytical and physical tasks (12, 13). Recently, artificial intelligence has been widely applied in EGD with the advancement in deep learning, which enables machines to reach human-like performance in many complex cognitive tasks (12, 14). Deep reinforced learning, which combines deep learning and reinforcement learning, has a stronger perception and decision-making ability to solve dynamic decision problems (15, 16). In our previous study, we have constructed a real-time artificial intelligence quality improving system based on deep reinforced learning which could monitor blind spots and assist diagnosis of high-risk lesions during EGD (10, 17–19).

In the present research, extending our previous study of the EGD artificial intelligence system, we investigated its efficacy on EGD training in the prospective randomized control trial.

## Methods

### Development of Computer-Aided (CAD) System

The CAD system was updated from the model classifying 26 anatomical landmarks of the upper digestive tract in our previous work (10). For the training and validation of the CAD, 75,742 qualified images (with more than 2,000 images each site) were used and 2,160 qualified images (with 80 images each site) were used for testing. The training and validation datasets were randomly separated in a ratio of 9:1. Google's TensorFlow deep learning framework was used for training, validation, and testing. ResNet-50 (20, 21) achieved an overall accuracy of 93.1% in the test dataset. Supplementary Table 1 shows the training images distribution in each site. The training, validation, and test datasets did not contain images from the same patient. The 26 anatomical landmarks of the upper digestive tract were shown in Supplementary Figure 1. All the images were retrospectively collected from five hospitals in China, including Renmin Hospital of Wuhan University, Wuhan; The First People's Hospital of Yichang, Yichang; Tongji Hospital, Tongji Medical College, Huazhong University of Science and Technology, Wuhan; Central Hospital of Wuhan, Wuhan; Yichang Central People's Hospital, Yichang.

**Participants**: In this study, six novice trainees without EGD experience from the Renmin Hospital of Wuhan University and Xiaogan Chinese Medical Hospital participated in the training program from December 24, 2020, to April 29, 2021. Inclusion criteria were as follows: (1) men or women who are over 18 years old; (2) trainees who have registered and obtained the practicing medical certification in China. Exclusion criteria were as follows: (1) trainees without qualified medical education certification; (2) trainees who refused to participate in clinical trials. Consecutive patients aged 18–75 years who were able to give informed consent were recruited. The exclusion criteria included patients who participated in other clinical trials, signed the informed consent form and within the follow-up period of other clinical trials; abused drugs or alcohol or mental disorders in the last 5 years; women who are pregnant or breastfeeding; patients with the previous history of gastric surgery; patients with high-risk diseases or other special conditions that are not suitable for participating in clinical trials; patients who declined to participate in the study. Each involved patient has approved one EGD procedure conducted by novice endoscopists and a second EGD conducted by senior endoscopists. The flowchart of patient recruitment was shown in Supplementary Figure 3.

### Trial Design

#### Pre- and Post-intervention Tests

**Figure 1:**
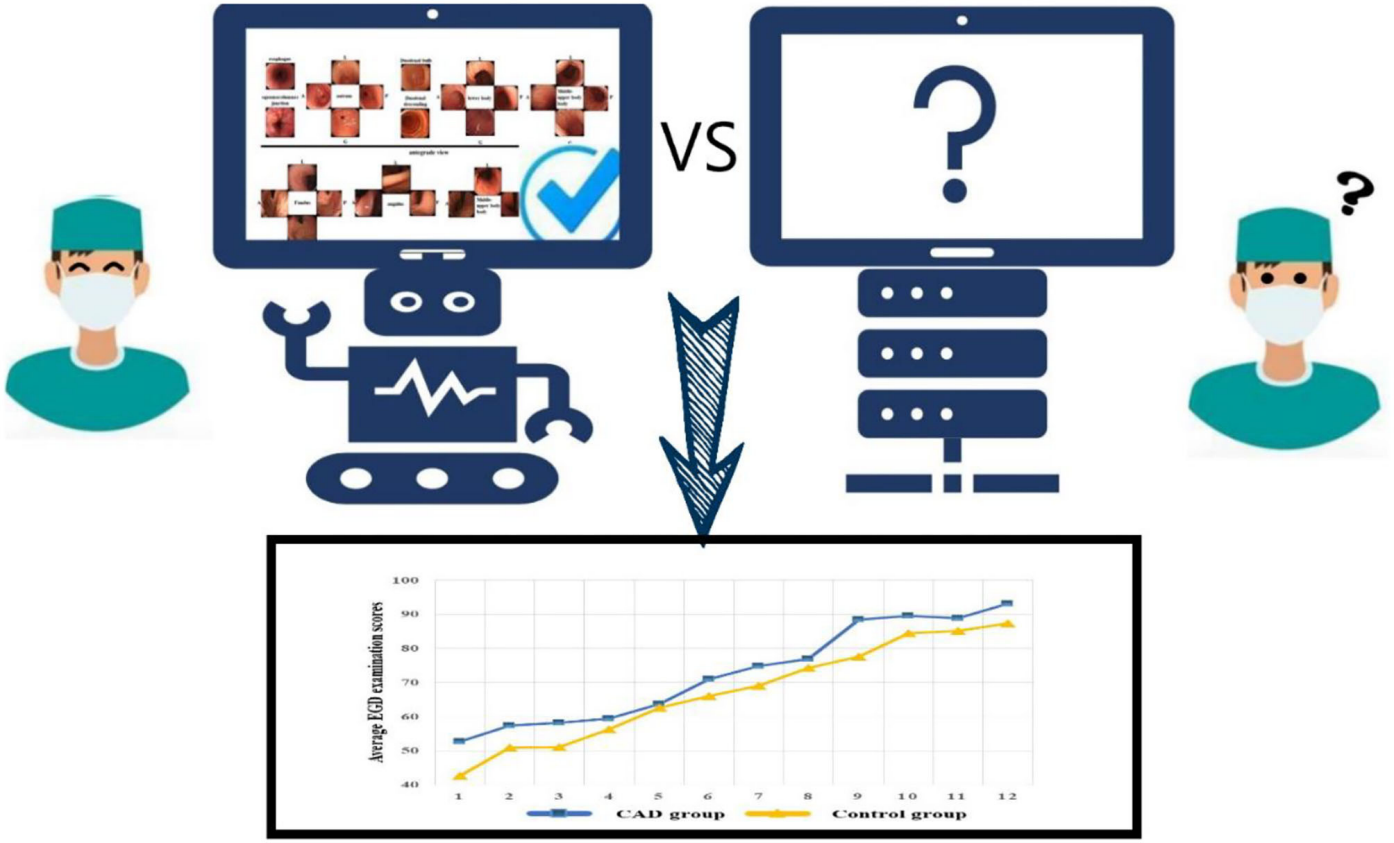
Graphic abstract of the study.

A parallel, randomized study was conducted in the Renmin Hospital of Wuhan University, and the allocation ratio was 1:1. Figure 1 is the graphic abstract of the study. The participants completed a pre-test before the training procedure, and a post-test 1 week after the whole training. The test, which was prepared by two experts from the Renmin Hospital of Wuhan University, included 50 one-choice questions, each of which was assigned two points and attached four to five options. The questions mainly focused on assessing the trainees' level of operating skills, identifying anatomical structures and common lesions of the upper gastrointestinal tract (UGI). The images used in the test were obtained from the Renmin Hospital of Wuhan University and approved by the ethics committee of the Renmin Hospital of Wuhan University. The pre-test and post-test consisted of the same questions but differed in order. After the pre-test, the six trainees were divided into two groups according to their test scores to guarantee the uniformity in the initial EGD performance of the two groups. The baseline characteristics of the patients were collected. The baseline characteristic of the trainees and their test scores are shown in Table 1.

**Table 1:** Baseline characteristics of trainees.

**Characteristics**	**Trainee 1**	**Trainee 2**	**Trainee 3**	**Trainee 4**	**Trainee 5**	**Trainee 6**
Group	CAD group	CAD group	CAD group	Control group	Control group	Control group
Age	27	26	27	26	27	34
Male/Female	Female	Male	Male	Female	Female	Male
Pre-test scores	60	58	52	52	62	56
Post-test scores	90	94	90	88	92	90
Previous EGD experience	No	No	No	No	No	No

**Randomization**: The two trainee groups selected covered grouping envelopes, and were randomly assigned to the CAD group and the control group. The patients that went for analgesic EGD were randomly assigned to either the CAD group or the control group. The random allocation sequence was a covered random envelope offered by the nurses.

#### EGD Examination Scoring

**Figure 2:**
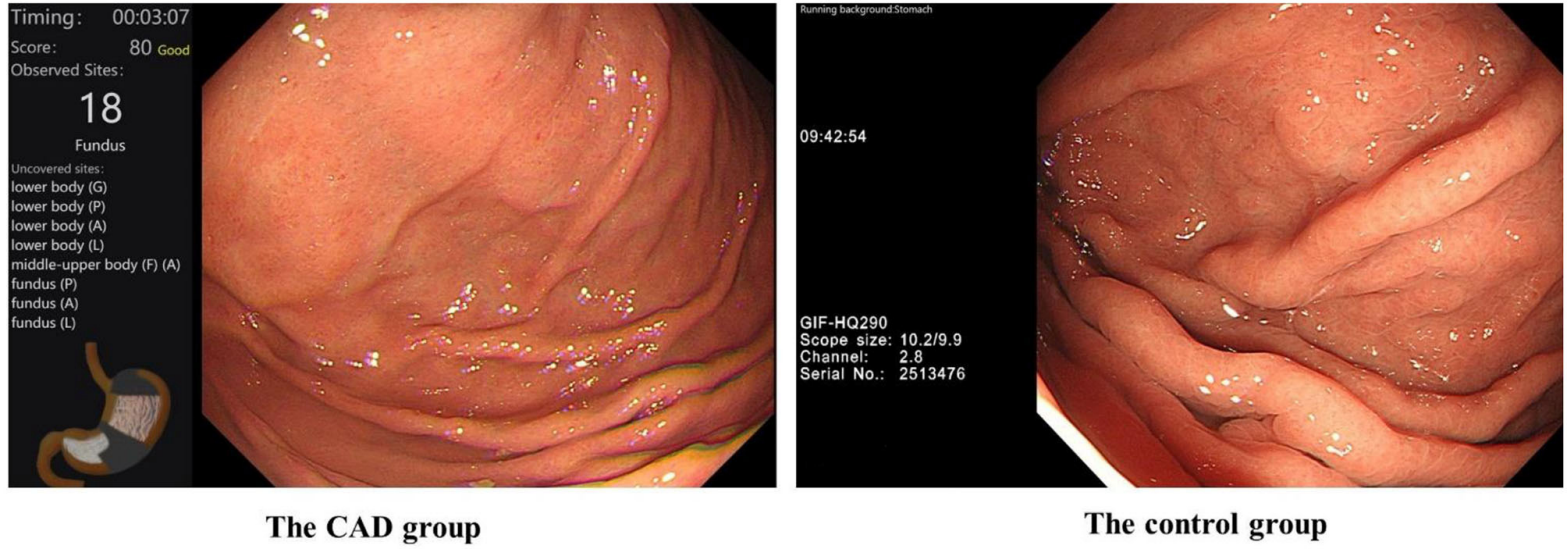
The display screen of the CAD group and control group during their training of EGD. #In the CAD group, the CAD system would remind the trainees of blind spots in real-time.

First, all the trainees completed a pre-test. Then, the trainees completed a 1 week training course together. During the course, they have taken the theory on EGD and they all have tried 10 EGD with the help of the senior endoscopists. The training enabled them to be competent for complete EGD procedures, then EGD learning and examination training began in two examination rooms under the instruction and supervision of two experienced endoscopists. In the CAD group, all the trainees would receive assistance from the CAD system in addition to the trainers' instruction. The control group was only trained with traditional methods by trainers. The display screens of the CAD group and control group during EGD are shown in Figure 2. The CAD group and control group exchanged the examination rooms and trainers (the two experienced endoscopists) every week. The patients would go through a second EGD operated by the endoscopists after the trainees' examination.

Two experts who were unaware of the grouping scored the trainees' EGD performance. When the scoring procedure began, only the screen that displayed the routine EGD video would be provided to them and the screen of the CAD system was turned back to the experts. We have referenced Li's research about the scoring scale applied in the examination, which includes operation skills (grade item 1, 35 points in total), withdrawal observation of mucosa (grade item 2, 35 points in total), overall examination time, and fluency (grade item 3, 5 points), comfort and satisfaction of patients (grade item 4, 5 points), position and definition of collected image (grade item 5, 10 points), and the diagnostic accuracy of lesions (grade item 6, 10 points) (8). The scoring scale was provided in Supplementary Material 2. In the scoring scale, the withdrawal observation of mucosa and diagnostic accuracy of lesions belong to recognition skills. Motor skills include gastroscopy forward operation, overall gastroscopy examination time and fluency, and comfort and satisfaction of patients. Qualified position and definition of the collected image need both motor and recognition skills. The gastroscopes used in the trial were from the vendor: Olympus Optical.

**Figure 3:**
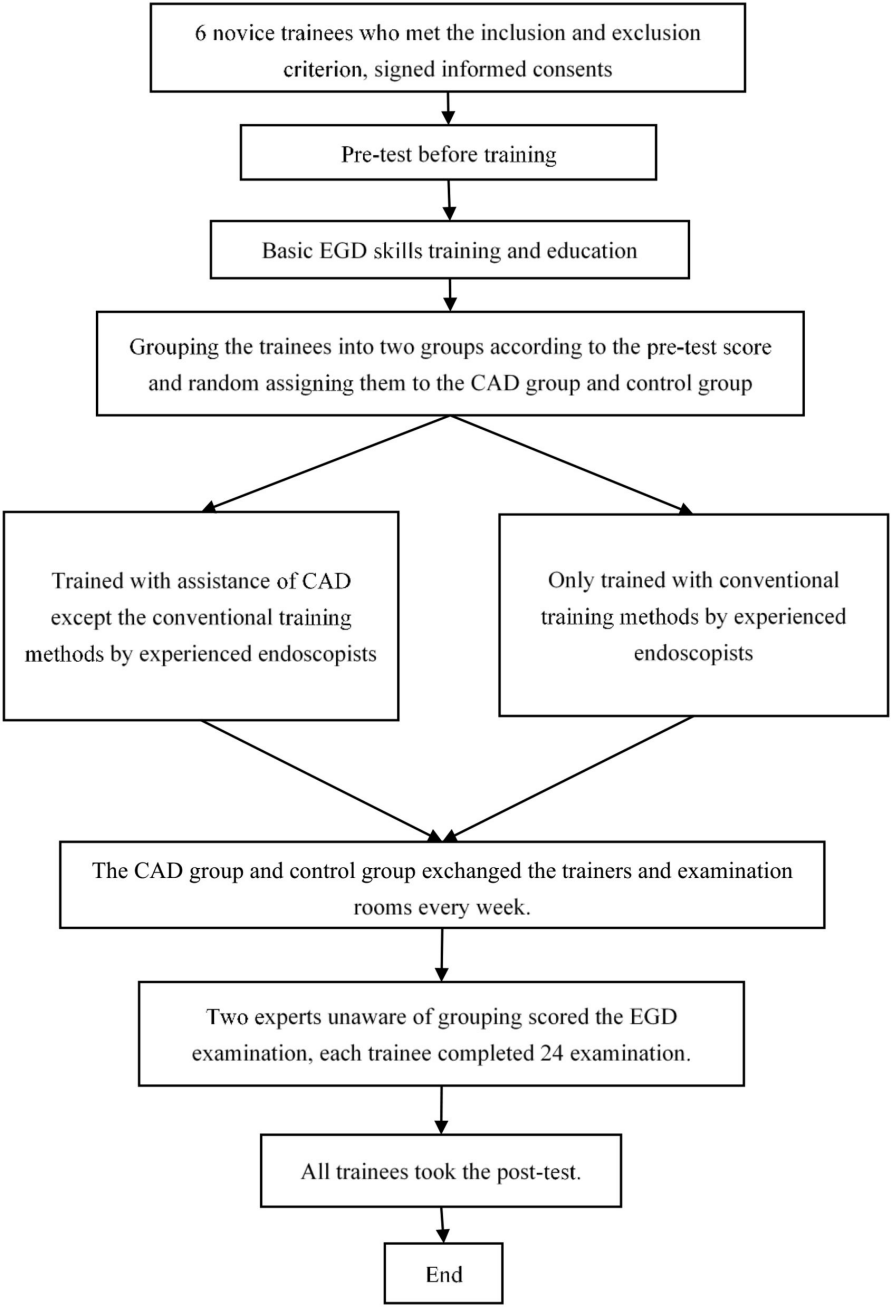
Flow chart of the study.

In the study, not all the EGD procedures conducted by the trainees were graded. In the clinical environment, lots of unexpected situations occur, such as patients with cough during anesthesia and changes in vital signs. Therefore, during the training procedure, the senior endoscopists sometimes had to terminate trainees and continue the EGD in case of adverse events. Referring to previous studies, the highest degree of completion trainees thought every two procedures for inclusion, in the final analysis, was chosen in this study to decrease the effect of the heterogeneity of individuals in the study. The flowchart of the study is shown in Figure 3.

#### Learning Curve

To visualize the competence over the training program, we built the learning curves of the CAD and control group. The learning curves were built with the average EGD examination scores, and the average scores were calculated every six procedures of each group to reduce individual differences and build a smooth learning curve. We defined every six procedures and one “stage” and the average scores of every six procedures of each group as “average EGD examination scores.” The learning curve was plotted with the “average EGD examination scores” as Y-axis and the “stage” as X-axis.

### Training Satisfaction

After the training, the trainees of the CAD group completed a satisfactory questionnaire about the CAD system (Supplementary Material 3). Each question contains five options. (Strongly agree = 5; agree = 4; Neutral = 3; Disagree = 2; Strongly disagree = 1).

### The Detection Rate of Lesions

The diagnosis of lesions was divided into polyp, ulcer, erosion, atrophy, esophagus lesions, and others. Taking the diagnosis of senior endoscopists as the golden standard, we defined the detection rate of the lesions as the proportion of lesion diagnosis by the trainees in lesions approved by the senior endoscopists.

**Outcomes**: The primary outcome was the EGD examination scores the trainees obtained. The secondary outcomes include the pre-test and post-test scores, the average blind spot rate (number of unobserved sites in each patient/26) in the two groups.

**Registration**: This trial is registered at ClinicalTrials.gov, number NCT04682821.

**Sample Size**: The study was intended to investigate the difference in EGD examination scores with or without the CAD system. According to previous literature, we estimated that the CAD group obtained five more grades than the control group at a 5% significance level and 80% power using a two-sided Student *t*-test. Assuming a 10% drop-out, a sample of 142 patients was needed. Sample size calculation was done using the software PASS 15.

### Statistical Analysis

The baseline characteristics were analyzed by chi-square test and student's *t*-test. The EGD examination scores were compared using the Mann-Whitney *U*-test. The blind spot rates of single sites were compared by the chi-square test. A Student's *t*-test was used for comparing the overall blind spot rate. All the data analysis was done by IBM SPSS version 26 (IBM, Armonk, New York, United States). The researchers who conducted the statistical analysis were unaware of the grouping.

## Results

From December 24, 2020, to April 29, 2021, six trainees without experience were recruited and all participated in the training program. They completed 288 EGD procedures and 144 were evaluated in the Renmin Hospital of Wuhan University. The study ended when the sample size was completed. The baseline characteristics of the patients in the two groups have no statistical differences (Table 2).

**Table 2:** Baseline characteristics of patients.

**Characteristics**	**CAD group (72)**	**Control group (72)**	***P*-value**
Age, mean (SD)	50.36 (13.26)	46.03 (13.22)	0.077
Female, *n* (%)	39 (54.17)	46 (63.89)	0.309
**Recruitment**, ***n*** **(%)**
Inpatient	7 (9.72)	5 (6.94)	
Outpatient	65 (90.28)	67 (93.06)	0.764
**Biopsy**, ***n*** **(%)**
Yes	56	61	0.393
No	16	11	

Compared with the pre-test, the average scores of the post-test increased significantly in both groups (CAD: 54.66 vs. 90.67; control group: 54.66 vs. 90.00). The CAD group demonstrated a higher level of competitiveness in the EGD examination compared with the control group (72.83 vs. 67.26), especially in the withdrawal observation of mucosa (26.40 vs. 24.11), overall examination time and fluency (3.75 vs. 3.42), and quality of collected images (7.29 vs. 6.70). There was no difference in operation skills, comfort and satisfaction of patients, and the diagnostic accuracy of lesions of the two groups. The average scores of each grading item are shown in Table 3.

**Table 3:** Primary outcome.

**Scoring list**	**CAD group, mean (SD)**	**Control group, mean (SD)**	***P*-value**
Overall scores	72.83 (16.12)	67.26 (15.64)	0.039
Operation skills	24.14 (7.20)	21.94 (6.62)	0.079
Withdrawal observation of mucosa	26.40 (6.13)	24.11 (6.21)	0.020
Overall examination time and fluency	3.75 (0.80)	3.42 (0.83)	0.034
Comfort and satisfaction of patients	4.13 (0.78)	4.03 (0.90)	0.702
Position and definition of collected image	7.29 (1.09)	6.70 (1.05)	0.006
Diagnostic accuracy of lesions	7.32 (0.99)	7.06 (1.21)	0.427

**Figure 4:**
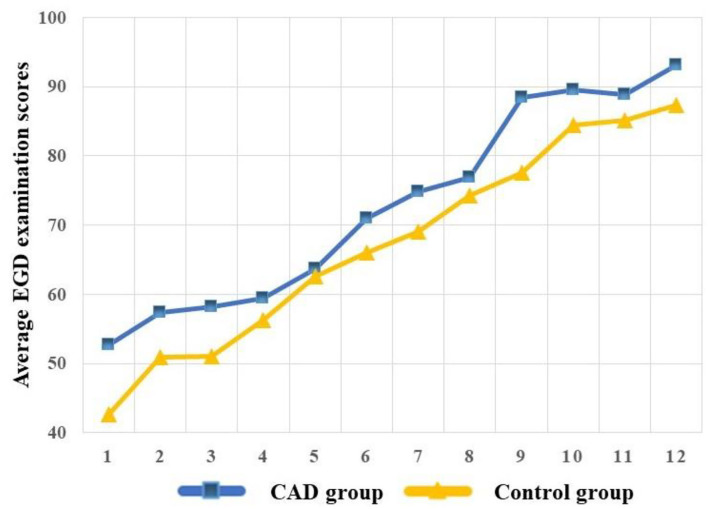
The learning curve of the CAD and control group.

As experience increased, the learning curve demonstrated measurable and significant improvement in the EGD examination scores of the two groups. The CAD group acquired higher competence compared with the control group (Figure 4).

During the whole training procedure, the CAD group completed the EGD examination with a lower blind spot rate than the control group (2.19 vs. 3.92%), especially in the Middle-upper body (R, L), Angulus (A), and Angulus (L) (Table 4).

**Table 4:** The blind spot rate of the computer-aided design (CAD) group and control group.

**Blind spot rate**	**CAD group**	**Control group**	**Odds ratio (95% CI^#^)**	***P*-value**
Overall, mean (SD)	2.19 (2.28)	3.92 (3.30)	NA	0.008
Esophageal (*n*, %)	0 (0.00)	0 (0.00)	NA	NA
Squamocolumnar junction (*n*, %)	0 (0.00)	0 (0.00)	NA	NA
Antrum (G) (*n*, %)	0 (0.00)	0 (0.00)	NA	NA
Antrum (P) (*n*, %)	2 (2.78)	2 (2.78)	1.00 (0.13–7.30)	1.00
Antrum (A) (*n*, %)	0 (0.00)	0 (0.00)	NA	NA
Antrum (L) (*n*, %)	0 (0.00)	0 (0.00)	NA	NA
Duodenal bulb (*n*, %)	3 (4.17)	6 (8.33)	0.48 (0.12–2.00)	0.31
Duodenal descending (*n*, %)	3 (4.17)	7 (9.72)	0.40 (0.10–1.63)	0.19
Lower body (G) (*n*, %)	5 (6.94)	4 (5.56)	1.27 (0.326–4.93)	0.73
Lower body (P) (*n*, %)	18 (25.00)	27 (37.50)	0.56 (0.27–1.14)	0.11
Lower body (A) (*n*, %)	9 (12.50)	12 (16.67)	0.71 (0.28–1.82)	0.48
Lower body (L) (*n*, %)	15 (20.83)	17 (23.61)	0.85 (0.39–1.87)	0.69
Middle-upper (G) (*n*, %)	9 (12.50)	16 (22.22)	0.50 (0.21–1.22)	0.12
Middle-upper (P) (*n*, %)	11 (15.28)	19 (26.39)	0.50 (0.22–1.15)	0.10
Middle-upper (A) (*n*, %)	11(15.28)	19 (26.39)	0.50 (0.22–1.15)	0.10
Middle-upper (L) (*n*, %)	11(15.28)	19 (26.39)	0.50 (0.22–1.15)	0.10
Fundus (G) (*n*, %)	6 (8.33)	8 (11.11)	0.73 (0.24–2.21)	0.57
Fundus (P) (*n*, %)	14 (19.44)	23 (31.94)	0.51 (0.24–1.11)	0.58
Fundus (A) (*n*, %)	3 (4.17)	7 (9.72)	0.40 (0.10–1.63)	0.19
Fundus (L) (*n*, %)	11(15.28)	18 (25.00)	0.54 (0.24–1.25)	0.15
Middle-upper body (R, P) (*n*, %)	7 (9.72)	14 (19.44)	0.45 (0.17–1.18)	0.10
Middle-upper body (R, A) (*n*, %)	7 (9.72)	11 (15.28)	0.60 (0.22–1.64)	0.31
Middle-upper body (R, L) (*n*, %)	4 (5.56)	19 (26.39)	0.16 (0.05–0.51)	0.001
Angulus (P) (*n*, %)	3 (4.17)	8 (11.11)	0.35 (0.09–1.37)	0.12
Angulus (A) (*n*, %)	0 (0.00)	8 (11.11)	1.13 (1.04–1.22)	0.004
Angulus (L) (*n*, %)	6 (8.33)	18 (25.00)	0.27 (0.10–0.74)	0.007

# *95% CI: 95% confidence interval*.

The training satisfactory scores of the trainees in the CAD group were 50, 48, and 48 points, respectively.

In Table 5, one case of polyp, one case of erosion, and one case of others were missed by the trainees of the CAD group. The other case is a xanthelasma on the gastric fundus. For the control group, two cases of polyp and three cases of erosion were missed. Among the three erosion cases, one was diagnosed as low-grade intraepithelial neoplasia by pathology. The five detection rates of the lesions demonstrated no statistical difference.

**Table 5:** Detection rate of different lesions.

**Lesions**	**The CAD group**	**The control group**	***P*-value**
Polyp	95.45% (21/22)	91.30% (21/23)	0.46
Ulcer	100% (5/5)	100% (4/4)	NA
Erosion	98.33% (59/60)	95.52% (64/67)	0.19
Atrophy	100% (23/23)	100% (14/14)	NA
Esophagus lesions	100% (20/20)	100% (16/16)	NA
Others	91.67% (11/12)	100% (7/7)	NA

## Discussion

Changes in the medical training environment, which greatly emphasize the patients' safety, effectiveness, and quality of endoscopy procedures, are in the quest for practical tools of EGD training. Various researchers made attempts to enhance the competence among trainees through *in vitro* simulators, centralized feedback systems, and so on, which were validated in various training programs (2–7, 9, 11, 22–24). While the motor skills can be rapidly acquired, the quality of EGD varies widely, and the unacceptably high rate of cancer misdiagnosis has arisen at endoscopy every year (1, 25). Guidelines from the European Society of Gastrointestinal Endoscopy (ESGE), the British Society of Gastroenterology (BSG), the Association of Upper Gastrointestinal Surgeons of Great Britain and Ireland (AUGIS), and the American Society for Gastrointestinal Endoscopy (ASGE) all have pointed out that a complete EGD should assess all relevant anatomical landmarks and high-risk stations with relevant photo-documentation and clear mucosa visualization (25–27). In the study, we have investigated the effect of the CAD system which can automatically recognize 26 anatomical landmarks of the upper digestive tract and document the relevant standard photos during EGD training of trainees without experience. The CAD group acquired a lower blind spot rate and more complete mucosa observation with comparable technical competence than the control group.

Qualified EGD requires motor and cognitive skills, and the clear mucosa observation can partly represent the motor skill level. Senior fellows can manipulate gastroscopes more smoothly and steadily with less blurred images during the EGD. In our study, the two groups are competent in motor skills, which can be speculated from the statistically indifferent average scores in the operation skills, comfort, and satisfaction of patients. These grade items are close with the technical competence and the similar outcomes can be attributed to the same training environment, procedures, and trainers the trainees received.

The blind spot rate is a useful quality descriptor to help define competence among trainees learning EGD and was validated in randomized control trials previously (3, 10, 18). The comprehensive observation of the upper digestive tract mucosa without blind spots can also be categorized into cognitive skills and be a quantitative quality indicator. The CAD group achieved 5.57 more scores averagely than the control group in the overall scores but those were the cognitive skills that contributed a lot to the difference. In the present study, the CAD group achieved a 1.73% lower blind spot rate than the control group. However, in Wu's study, the blind spot rate was 16.6% higher in the control group than the CAD-assisted group on average. The EGD blind spot rate in our previous study is more distinct than this study. We thought that two main factors led to the difference. First, the previous study recruited skilled endoscopists while in the present study all the participants were novice trainees, who knew they were surveilled and tested. They could be highly concentrated on every procedure they completed. Second, the sample size adopted in this study was calculated according to the primary endpoint, EGD examination scores, rather than the blind spot rate, which was used as the primary endpoint in Wu's study. We were convinced that the more distinct difference of blind spot rate would appear with the increase of sample size.

The two groups acquired a relatively parallel learning curve with a different starting point. The CAD group was assisted with CAD at the beginning of training so they could get high scores on the mucosa observation item of the grading scale initially while the control group did not. Their motor skills improved equivalently as experience increased. The detection rate of lesions has no statistical difference between the two groups. In addition to the aforementioned endpoints, the trainees felt much more reassured and confident with less worry about the blind spot or potentially missed lesions when trained with the CAD which enabled them to achieve more complete observation rather than repeat the procedure only by memories. The CAD group usually spent a long time observing the mucosa.

The Assessment of Competency in Endoscopy (ACE) tools for EGD was put forward by the Training Committee of ASGE, which consisted of seven questions to evaluate specific motor and cognitive skills and two additional questions assessing overall motor and cognitive ability (28–30). Acting as comparative standards, the ACE tools are highly generalized and can be easily influenced by subjective consciousness, which also has been pointed in other studies (3). The gastroscopy direct observation of procedural skills (DOPS) for Diagnostic upper gastrointestinal endoscopy (OGD), which is assessed centrally to determine whether a trainee should receive certification for independent EGD, is a formative assessment tool administered by the Joint Advisory Group on Gastrointestinal Endoscopy (JAG) (31). DOPS-OGD covers the assessment of integrated EGD procedure including pre-procedure preparation, endoscope insertion and withdrawal, mucosa visualization, management of findings, post-procedure management, and endoscopic non-technical skills, and each domain contains detailed items. In this study, we have adopted Li's grading table to evaluate the competence of trainees. This grading table is detailed in each item like the DOPS-OGD. But compared with the DOPS-OGD, it concentrates more on endoscope insertion and withdrawal, mucosa visualization, and management of findings without assessing pre-procedure preparation and post-procedure management. In the healthcare center where this study was carried out, the pre-procedure preparation and post-procedure management are conducted by physicians and primary nurses, and anesthesiologists are responsible for sedation. Li's grading table was more suitable and ultimately chosen for the EGD training assessment. The pre-procedure preparation and post-procedure management are certainly vital components of EGD training assessment. We should apply more completed assessment tools like DOPS-OGD to validate the efficacy of the CAD system in the future.

There are some limitations to our study. As a single-center study, the efficacy of the CAD system needs enlarged validation in more healthcare centers. The number of participants in this study is limited; however, we have adopted the pre-test for grouping to reduce the possible systematic error. Even though all the trainees completed the training program and were capable to perform qualified EGD, they still need more practice to reinforce their skills to be more professional. For the CAD group, there might be some potential distraction of the trainees with the blind spots reminding of the CAD. The circumstance that the trainees concentrated more on whether the landmarks were checked or not than scrutinizing the mucosa cannot be excluded. To prevent the potential distraction, we emphasize the self-inspection of the CAD when first introducing the system to the novice. The trainees could find the blind spots when the procedure is mainly completed rather than throughout the procedure. In the future, we would enlarge our study in multiple centers and recruit more trainees to validate the efficacy of the CAD system.

In the present study, we have investigated the effectiveness of the AI assistant system on EGD training for novice trainees. Compared with the control group, the CAD group achieved higher EGD examination scores and a lower blind spot rate. The AI assistant system displayed exceptional capacity on standard EGD training and assisted the trainees in achieving a faster learning curve and higher operation quality.

## Data Availability Statement

The raw data supporting the conclusions of this article will be made available by the authors, without undue reservation.

## Ethics Statement

The studies involving human participants were reviewed and approved by the Ethics Committee of Renmin Hospital of Wuhan University. The patients/participants provided their written informed consent to participate in this study.

## Author Contributions

HY, LW, and JL designed the study. YX, JL, and QX conducted the literature search. MX, LY, LZ, MZ, DW, and RS completed the data collection. YZ and DG performed the data analysis. LH wrote the paper. All authors contributed to the article and approved the submitted version.

## Funding

Hubei Provincial Clinical Research Center for Digestive Disease Minimally Invasive Incision (Grant No. 2018BCC337), Hubei Province Major Science and Technology Innovation Project (Grant No. 2018-916-000-008).

## Conflict of Interest

The authors declare that the research was conducted in the absence of any commercial or financial relationships that could be construed as a potential conflict of interest.

## Publisher's Note

All claims expressed in this article are solely those of the authors and do not necessarily represent those of their affiliated organizations, or those of the publisher, the editors and the reviewers. Any product that may be evaluated in this article, or claim that may be made by its manufacturer, is not guaranteed or endorsed by the publisher.
