# Supplementary material for: Impact of Computer-Assisted System on the Learning Curve and Quality in Esophagogastroduodenoscopy: Randomized Controlled Trial
Source: Front Med (Lausanne). 2021 Dec 14;8:781256. doi: 10.3389/fmed.2021.781256 (PMC8713729; doi:10.3389/fmed.2021.781256)
Supplement: Supplementary file 1 [file Table_2.DOCX]

**Supplementary materials 2:**

**Grading Table for Gastroscopy Examination**

| **Candidate number：** | |  | **Score：** | |  |  |  |  |
| --- | --- | --- | --- | --- | --- | --- | --- | --- |
|  |  |  |  |  |  |  |  |  |
|  | Examination contents | |  |  | Fraction | | Score |  |
|  |  | |  | |  |  |  |  |
| 1.Gastroscopy | Preparation before the examination, holding | | | | 5 |  |  |  |
| forward | gastroscope method | |  |  |  |  |  |  |
| operation |  | |  | |  |  |  |  |
|  | Operation from oropharyngeal to esophageal | | | | 5 |  |  |  |
|  | entrance | |  |  |  |  |  |  |
|  |  | |  |  |  |  |  |  |
|  | Operation through esophagus | |  |  | 5 |  |  |  |
|  |  | |  | |  |  |  |  |
|  | Entering the body of the stomach through the | | | | 5 |  |  |  |
|  | cardia and fundus of the stomach | | | |  |  |  |  |
|  |  | |  | |  |  |  |  |
|  | Operation through gastric body and antrum | | | | 5 |  |  |  |
|  |  | |  | |  |  |  |  |
|  | Access to the duodenum through the pylorus | | | | 5 |  |  |  |
|  |  | |  | |  |  |  |  |
|  | Entering the descending segment of the duodenum | | | | 5 |  |  |  |
|  | through the upper corner of the duodenum | | | |  |  |  |  |
|  |  | |  | |  |  |  |  |
| 2.Gastroscopy | Observation of descending segment of duodenum | | | | 5 |  |  |  |
| withdrawal |  | |  |  |  |  |  |  |
|  | Observation of duodenal bulb | |  |  | 5 |  |  |  |
| observation |  | |  |  |  |  |  |  |
|  | Observation of gastric antrum | |  |  | 5 |  |  |  |
|  |  | |  |  |  |  |  |  |
|  | Observation of gastric angle | |  |  | 5 |  |  |  |
|  |  | |  | |  |  |  |  |
|  | Observation on the fundus and cardia of stomach | | | | 5 |  |  |  |
|  |  | |  |  |  |  |  |  |
|  | Observation of gastric body | |  |  | 5 |  |  |  |
|  |  | |  | |  |  |  |  |
|  | Observation of esophagus and cardia | | | | 5 |  |  |  |
|  |  | |  | |  |  |  |  |
| 3. Overall gastroscopy examination time and fluency | | | | | 5 |  |  |  |
|  | | |  | |  |  |  |  |
| 4. Response, comfort and satisfaction of patients during | | | | | 5 |  |  |  |
| gastroscopy |  |  |  |  |  |  |  |  |
|  | | |  |  |  |  |  |  |
| 5. Position and definition of collected image | | |  |  | 10 |  |  |  |
|  | | |  |  |  |  |  |  |
| 6. Diagnostic accuracy under gastroscopy | | |  |  | 10 |  |  |  |
|  |  | |  |  |  |  |  |  |
|  | Total | |  |  | 100 |  |  |  |
|  | | |  |  |  |  |  |  |
| Errors or deficiencies of candidates: | | |  |  |  |  |  |  |
|  | | |  | |  |  |  |  |
| Signature of assessment expert: | | | Date: | |  |  |  |  |
|  |  |  |  |  |  |  |  |  |

[1]

1. Li, S., et al., *Development and assessment of a gastroscopy electronic learning system for primary learners: randomized controlled trial.* Journal of medical Internet research, 2020. **22**(3): p. e16233.
