# Supplementary material for: Impact of Computer-Assisted System on the Learning Curve and Quality in Esophagogastroduodenoscopy: Randomized Controlled Trial
Source: Front Med (Lausanne). 2021 Dec 14;8:781256. doi: 10.3389/fmed.2021.781256 (PMC8713729; doi:10.3389/fmed.2021.781256)
Supplement: Supplementary file 2 [file Table_3.DOCX]

**Supplementary material 3:**

**“Computer-aided system for EGD training” Satisfaction Questionnaire**

Please give your opinions and feedback after using the computer-aided system and fill in the selected items. Thanks for your devotion!

| **Survey Items** | **Survey Content** | **Satisfaction** | | | | |
| --- | --- | --- | --- | --- | --- | --- |
|  |  | Strongly agree | Agree | Neutral | Disagree | Strongly disagree |
| 1 | The CAD system provides useful content and fits my needs. |  |  |  |  |  |
| 2 | The CAD system is easy to use. |  |  |  |  |  |
| 3 | The display screen of the CAD system is easy to understand. |  |  |  |  |  |
| 4 | The video of the CAD system displayed on its screen is fluent. |  |  |  |  |  |
| 5 | The operation of the CAD system is stable. |  |  |  |  |  |
| 6 | The CAD system makes it easy for me to evaluate my learning performance. |  |  |  |  |  |
| 7 | The CAD system enables me to control my learning progress. |  |  |  |  |  |
| 8 | With the CAD system, I feel more confident. |  |  |  |  |  |
| 9 | Overall, you are satisfied with the CAD system. |  |  |  |  |  |
| 10 | Overall, the CAD system is successful. |  |  |  |  |  |
| **Suggestions and comments:** | | | | | | |
