# Supplementary material for: Impact of Computer-Assisted System on the Learning Curve and Quality in Esophagogastroduodenoscopy: Randomized Controlled Trial
Source: Front Med (Lausanne). 2021 Dec 14;8:781256. doi: 10.3389/fmed.2021.781256 (PMC8713729; doi:10.3389/fmed.2021.781256)
Supplement: Supplementary file 3 [file Presentation_1.PDF]

Esophagogastroduodenoscopy Test

(Time: 30 minutes)

Name:________________Date test taken:_______________Total marks：____________

Single Choice (Choose only one answer from the four/five choices as the most appropriate answer，50 questions * 2 marks each)

1. What is the most suitable position for patients undergoing routine gastroscopy? (   )

A. Left lateral recumbent position with flexion of both knees.

B. Right lateral decubitus with flexion of both knees.

C. Supine position, knees straight.

D. Left recumbent position with knees straight.

E. Prone position, knees straight.

2. During the gastroscopy, if an improper operation results in the reflex of gastroscopy in the esophagus, which of the following is the best reaction? (   )

A. Pull out the endoscope immediately.

B. Relieve the reflex immediately in the esophagus.

C. Insert the endoscope quickly into the stomach to relieve reflex in a wide stomach cavity.

D. Slowly introduce a curved endoscope body into the stomach to relieve reflex in a wide stomach cavity.

E. Leave in a panic.

3. Which is the contraindication to painless gastroscopy? (   )

A. Those who have gastroscopy indications but fear routine gastroscopy.

B. Severe nausea, vomiting, or other reasons are difficult to complete routine gastroscopy.

C. Snoring and obesity.

D. Voluntary request for painless gastroscopy.

E. Patients' age < 18 years.

4. During painless gastroscopy, the patient should be closely monitored (   )

A. Vital signs of patients, establish effective venous channels.

B. Keep the respiratory tract unobstructed.

C. Blood oxygen saturation ≤ 90%, pressurize oxygen immediately.

D. All of the above.

E. None of the above.

5. Which of the following is not the prerequisite work before a gastroscopy? (   )

A. Start fasting 12 hours before the inspection and drink water.

B. Abstain from drinking water and take antihypertensive drugs after going to bed on the day of inspection.

C. To inhibit gastric peristalsis and secretion of gastric juice, antispasmodics and sedatives can be selected 30 minutes before examination according to the situation.

D. Patients were given lidocaine glue 30 minutes before the operation
E. If the patient has a removable denture, it should be removed.

6. The three physiological strictures of the normal adult esophagus are about ( )

A. 10cm，15cm， 20cm
B. 15cm，25cm，40cm
C. 15cm，25cm，35cm
D. 10cm，25cm，40cm
E. 10cm, 25cm, 35cm

7. The second esophageal stricture is located in ( )

A. Aortic arch and tracheal bifurcation B. Upper edge of sternum C. Bifurcation of ascending aorta and organs D. Ring thyroid level E. About 30cm from incisor

8. The gastric ulcer usually occurs in ( )

A. Fundus B. Antrum C. Greater Curvature and Angle D. Smaller Curvature and Antrum E. Angle and Smaller Curvature

9. Which of the following are the typical lesions of Gastric erosion? ( )

A. Maximum diameter < 0.2cm.
B. Maximum diameter < 0.5cm.
C. Depth not exceeding muscle layer.
D. The depth does not exceed the muscularis mucosae.
E.The depth does not exceed the mucosal layer.

10. The critical trait of grade A endoscopic images of reflux esophagitis (Los Angeles) is ( ).

A. At least one fusion injury with more than two mucosal folds.
B. The length of mucosal lesions should not exceed 5 mm.
C. Whole-circle mucosal injury.
D. At least one mucosal lesion was more than 5 mm in diameter, but no fusion occurred.
E. Mucosal lesions are interconnected, exceeding 75% of the circumference of the esophagus.

11. Which of the following is the characteristic of stage S1 duodenal ulcer ( )

A. The bottom is covered with thick moss, and there are hemorrhagic spots or clots attached. The surrounding mucosa is markedly hyperemic, edematous, and erosive.

B. Peripheral hyperemia and edema of ulcer were alleviated, white moss was clean, the boundary was clear, and the concentration of mucosal folds was not obvious.

C. The ulcer shrank and became shallow, the white fur edge was smooth, the edema disappeared, the surrounding regenerated epithelium was obvious, and the mucosal fold concentrated sign appeared.

D. The ulcer was reduced, the white fur became thinner and the regenerated epithelium was widened.

E. All ulcers are covered by regenerated epithelium, which is red and arranged in grid-like centripetal radiation.

12. Which upper digestive tract structure is shown in the figure? ( )

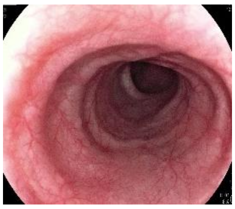

A. Esophagus

B. Cardia

C. Gastric body

D. Duodenal bulb

E. Descending duodenum

13. Which is the view of gastric angulus ?（ ）

A 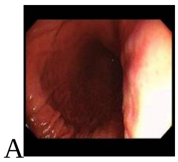 B. 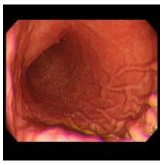 C. 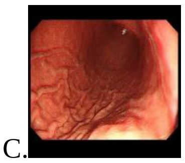 D. 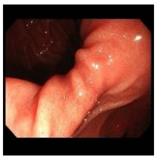 E. 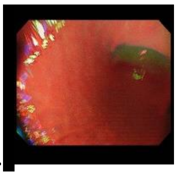

14. Which upper digestive tract structure is shown in the figure? ( )

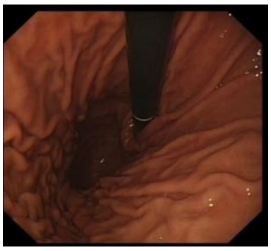

A．Gastric body B. Gastric angle C. Descending duodenum D. Duodenal bulb E. Cardia

15. Which is the retroflex view of the middle-upper gastric body？（）

A. 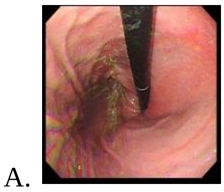 B. 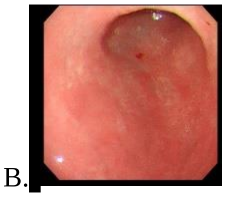 C. 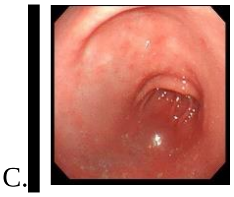 D. 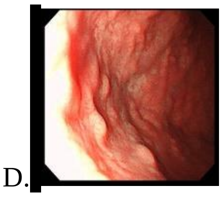 E. 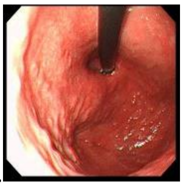

16. The diagnosis of the following endoscopic view could be ( )

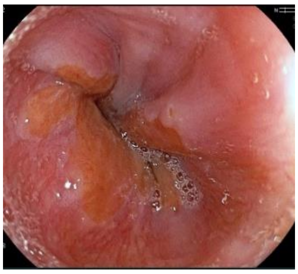

A. Reflux esophagitis B. Chronic superficial gastritis C. Duodenitis D. Normal esophagus E. Achalasia of cardia

17. Which of the following images can be diagnosed as esophageal papilloma ( )

A. 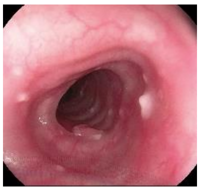 B. 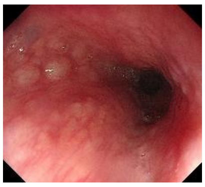 C. 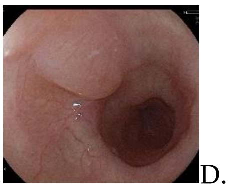 D. 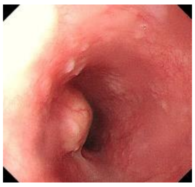

18. The diagnosis of the following endoscopic view could be（ ）

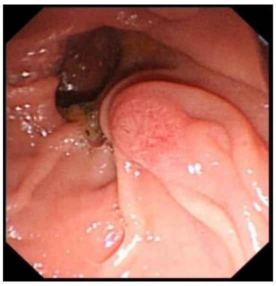

A. Duodenal lymphoma B. Duodenal lipoma C. Duodenal hemangioma D. Duodenal papilla E. Duodenal papilla carcinoma

19. Which of the following images can be diagnosed as infection of helicobacter pylori ( )

A. 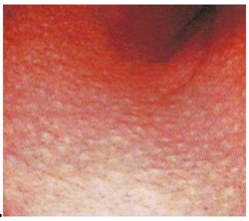
B. 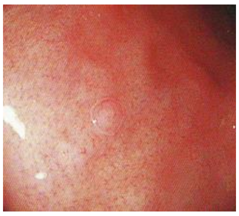
C. 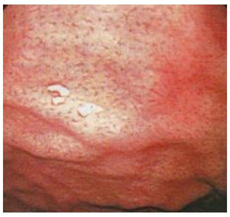
D. 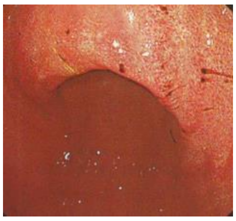

20. Endoscopic classification of early gastric cancer (*Japanese Endoscopic Classification*) is ( )

A. Uplift type, superficial type, and depression type

B. Superficial, protruding and infiltrating types

C. Surface type, infiltrating type, and mixed type

D. Surface type, fungus type, and depression type

E. Protuberant, superficial and infiltrative type

21. Which of the following images can be diagnosed as ulcer of A1 stage? ( )

A. 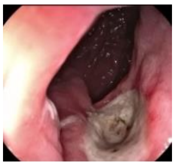
B. 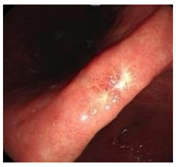
C. 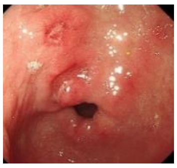
D. 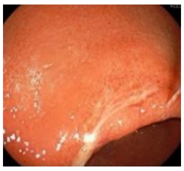
E. 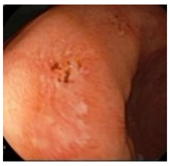

22. The diagnosis of the following endoscopic view could be ( )

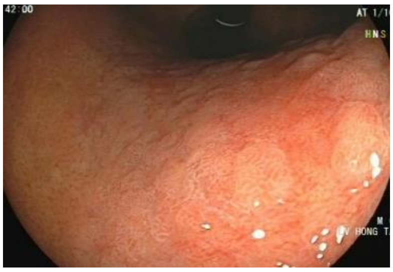

A. Non-atrophic gastritis

B. Atrophic gastritis (simple atrophy)

C. Atrophic gastritis (atrophy with intestinal metaplasia)

D. Low-grade intraepithelial neoplasia

E. High-grade intraepithelial neoplasia and early carcinoma

23. Which of the following images can be diagnosed as gastric cancer ( )

a 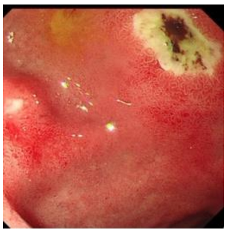 b 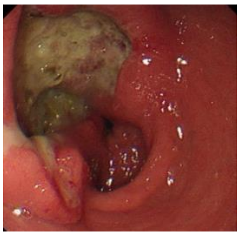

A. a and b.

B. a.

C. b.

D. Both a and b are not cancer.

E. Not sure.

24. In the following figures of early gastric cancer, which one belongs to 0-IIa+IIc of *Paris Classification*? (   )

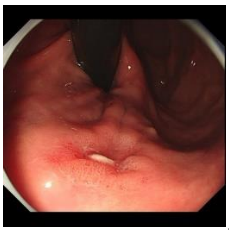 A 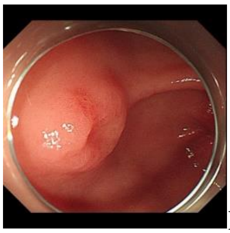 B 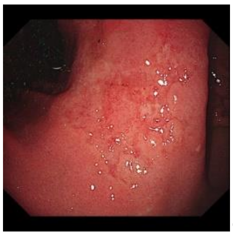 C 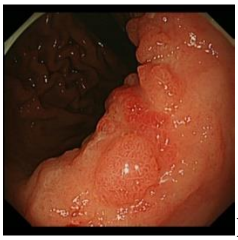 D

25. Which of the following is not classified as "submucosal tumors of the digestive tract"? (   )

A. Neuroendocrine tumor

B. Lipoma

C. Early gastric cancer

D. Ectopic pancreas

E. Leiomyoma

26. Which of the following figures is most close to "reflux esophagitis of grade D (*Los Angeles Classification*)"? (   )

A. 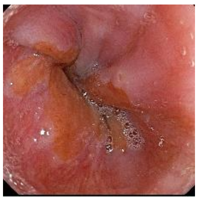 B. 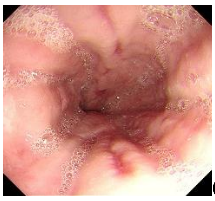 C. 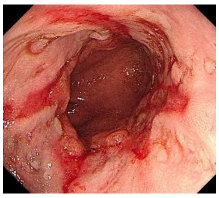 D. 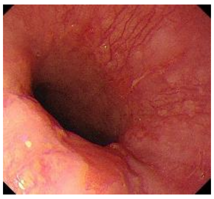

27. The diagnosis of the following endoscopic view could be (   )

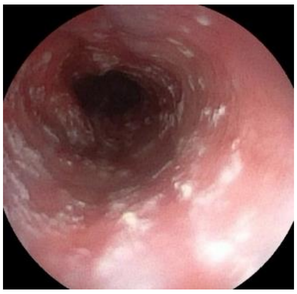

A. Reflux esophagitis B. Esophageal glycogen acanthosis C. Esophageal foreign body D. Esophageal cancer E. Fungal esophagitis

28. The diagnosis of the following endoscopic view could be (   )

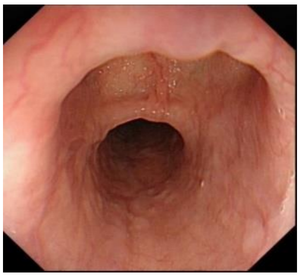

A. Esophageal polyps

B. Achalasia of cardia

C. Barrett's esophagus

D. Hiatal hernia of esophagus

E. Esophageal diverticulum

29. Which of the following figures is most close to “Mallory-Weiss Syndrome”? ( )

A. 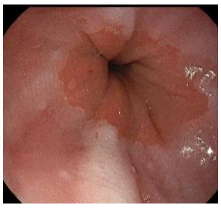 B. 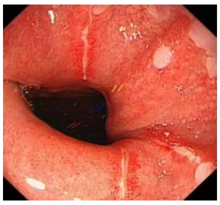 C. 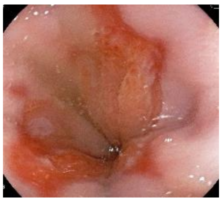 D. 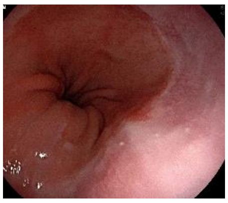

30. The diagnosis of the following endoscopic view could be (   )

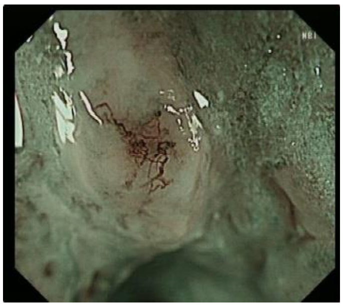

A. Early esophageal cancer SM1 B. Early esophageal cancer SM2 C. Early esophageal cancer SM3 D. Advanced esophageal cancer

31. In the following images, which one is most close to gastric lipoma ( )

A. 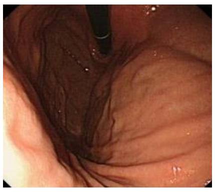 B. 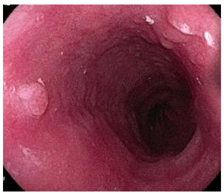 C. 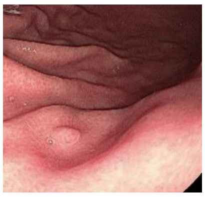 D. 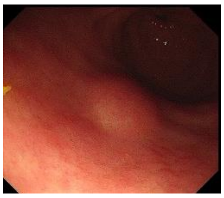

32. The diagnosis of the following endoscopic view could be ( )

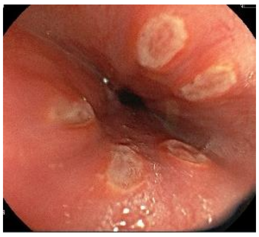

A. Reflux esophagitis

B. Esophageal heterotopic gastric mucosa

C. Barrett's esophagus

D. Behcet's disease

E. Glycogen echinoderm of esophagus

33. Which of the following figures is most close to achalasia? (   )

A. 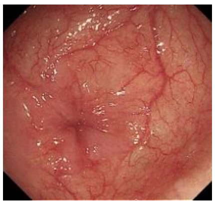 B. 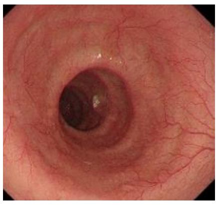 C. 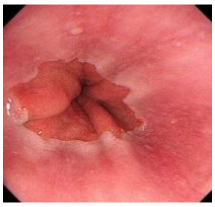 D. 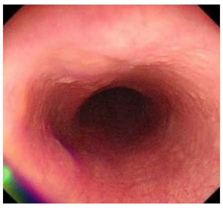

34. The diagnosis of the following endoscopic view could be (   )

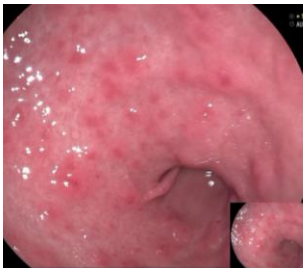

A. Erosive gastritis

B. Multiple gastric polyps

C. Portal hypertensive gastropathy

D. Hemorrhagic gastritis

E. Normal gastric mucosa

35. The diagnosis of the following endoscopic view could be (   )

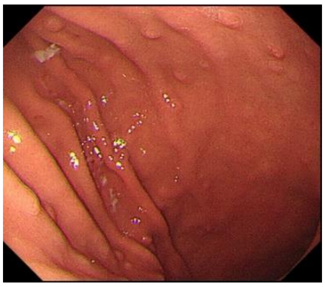

A. Acute gastritis B. Portal hypertensive gastropathy C. Gastric cancer D. Gastric ulcer E. Multiple gastric polyps

36. Which of the following images should not be classified as the Hemangioma of upper digestive tract? (   )

A. 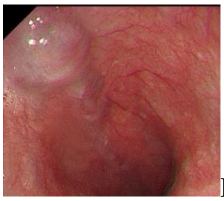 B. 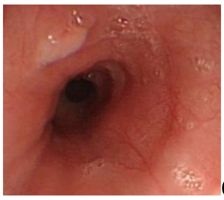 C. 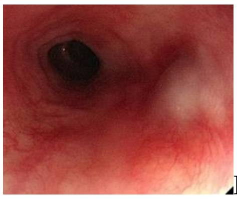 D. 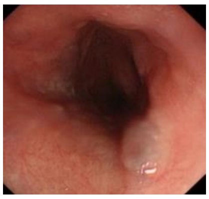

37. The diagnosis of the following endoscopic view could be (   )

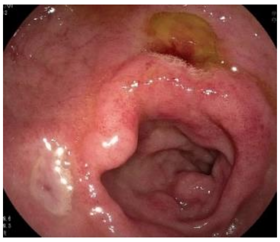

A. Gastric ulcer B. Duodenal ulcer C. Gastritis D. Gastric cancer E. Duodenal tumor

38. The diagnosis of the following endoscopic view could be ( )

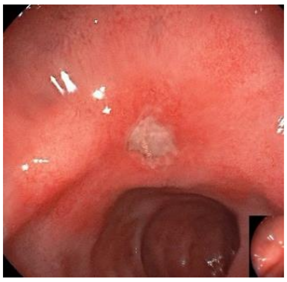

A. Gastric ulcer B. Duodenal bulbar ulcer C. Duodenal descending ulcer D. Duodenal tumor E. Malignant gastric ulcer

39. In the following endoscopic picture, which one is most close to "chronic atrophic gastritis"? ( )

A. 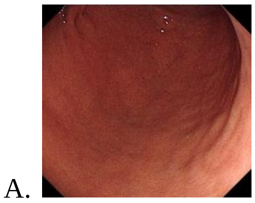 B. 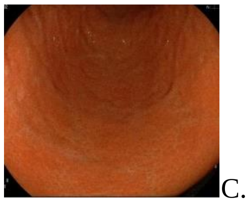 C. 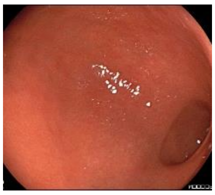 D. 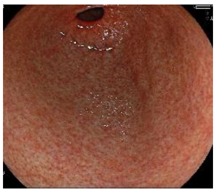

40. The diagnosis of the following endoscopic view could be ( )

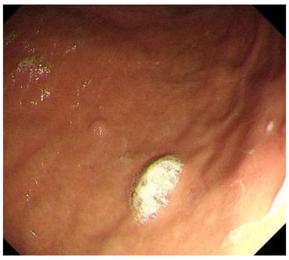

A. Gastric polyps B. Gastric hemangioma C. Gastric heterotopic pancreas D. Gastric xanthoma E. Gastric lipoma

41. The diagnosis of the following endoscopic view could be ( )

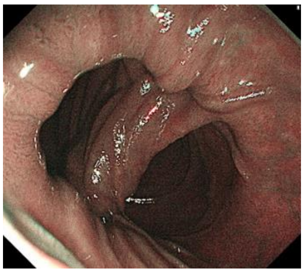

A. Postoperative gastric B. Duodenal diverticulum C. Esophageal diverticulum D. Esophageal-tracheal fistula E. Cardiac achalasia

42. The diagnosis of the following endoscopic view could be ( )

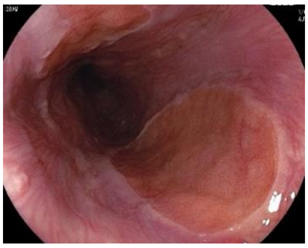

A. Ectopic gastric mucosa of esophagus B. Barrett's esophagus C. Reflux esophagitis D. Fungal

esophagitis E. Esophageal glycogen acanthosis

43. The diagnosis of the following endoscopic view could be ( )

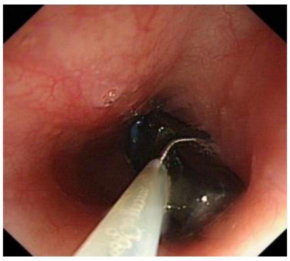

A. Gastric cancer B. Gastric papilloma C. Gastric foreign body D. Gastrolith E. Gastric ulcer

44. Which one of the following images may be diagnosed as early gastric cancer? （ ）

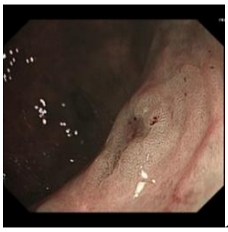 A 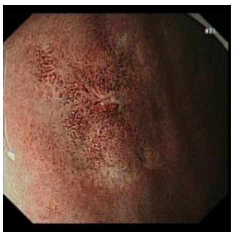 B 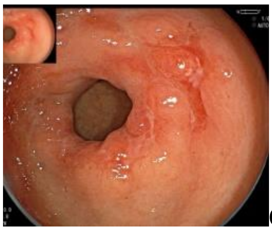 C 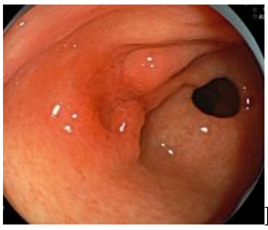 D

45. The diagnosis of the following endoscopic view could be ( )

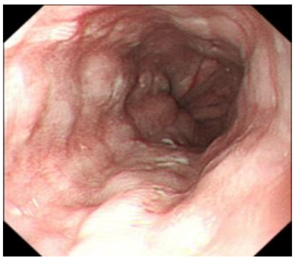

A. Esophageal polyps B. Esophageal varices C. Esophageal leiomyoma D. Esophageal papilloma

E. Esophageal venous aneurysm

46. The diagnosis of the following endoscopic view could be ( )

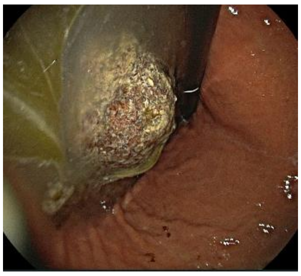

A. Gastric cancer B. Gastric ulcer C. Gastric cyst D. Gastric xanthelasma E. Gastric calculus

47. Which one of the following images can be diagnosed as intestinal metaplasia ( )

A. 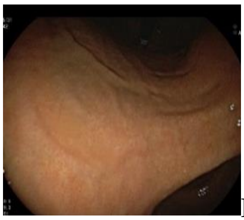 B. 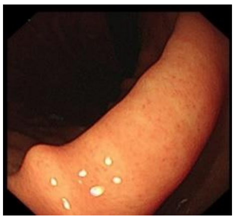 C. 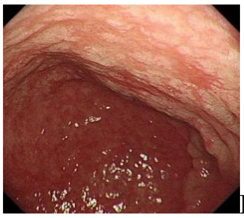 D. 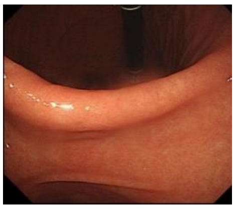

48. The diagnosis of the following endoscopic view could be ( )

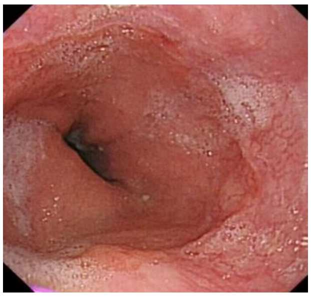

A. Achalasia B. Reflux esophagitis C. Esophageal and cardiac mucosal laceration D. Esophageal hiatal hernia E. Esophageal diverticulum

49. The diagnosis of the following endoscopic view could be (   )

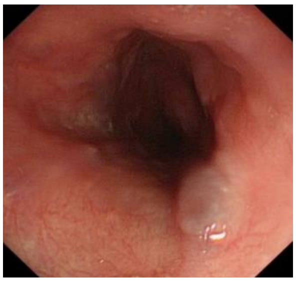

A. Esophageal polyps B. Esophageal varices C. Esophageal leiomyoma D. Esophageal papilloma E. Esophageal venous aneurysm

50. The diagnosis of the following endoscopic view could be (   )

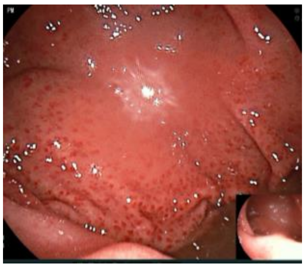

A. Erosive gastritis
B. Multiple gastric polyps
C. Portal hypertensive gastropathy
D. Hemorrhagic gastritis
E. Normal gastric mucosa
