# Supplementary material for: Impact of Computer-Assisted System on the Learning Curve and Quality in Esophagogastroduodenoscopy: Randomized Controlled Trial
Source: Front Med (Lausanne). 2021 Dec 14;8:781256. doi: 10.3389/fmed.2021.781256 (PMC8713729; doi:10.3389/fmed.2021.781256)
Supplement: Supplementary file 4 [file Data_Sheet_1.docx]

**Supplementary materials 1:**

**The construction of the CAD system.**

75742 qualified images (with more than 2000 images on each site) were used for training and validation of the CNN2 and 2160 qualified images (with 80 images in each site) for testing. The training, validation, and test sets didn’t contain images from the same patient. Training and validation datasets were randomly separated in a ratio of 9:1. K-fold cross-validation procedure was implemented with k=10, dividing the training dataset into 10 subsets and validating each subset individually with the remaining used for training^1^. Google’s TensorFlow deep learning framework was used for training, validation, and testing. ResNet-50^2^ achieved an overall accuracy of 93.1% in the test dataset after converging st the 18th epoch. In the supplementary Figure 1, the numbers represented: 0:unqualified images, 1: Esophageal, 2: Squamocolumnar junction, 3:Antrum (G), 4:Antrum (P), 5: Antrum (A), 6: Antrum (L), 7: Duodenal bulb, 8: Duodenal descending, 9: Lower body (G), 10: Lower body (P), 11: Lower body (A), 12: Lower body (L), 13: Middle-upper (G), 14: Middle-upper (P), 15: Middle-upper (A), 16: Middle-upper (L), 17: Fundus (G), 18: Fundus (P),19: Fundus (A), 20: Fundus (L), 21: Middle-upper body (R, P), 22: Middle-upper body (R, A), 23: Middle-upper body (R, L), 24: Angulus (P), 25: Angulus (A), 26: Angulus (L). The column is the true label of the images in the test dataset and the row is the label CAD predicted.

1. Wen Z, Li B, Kotagiri R, Chen J, Chen Y, Zhang R. Improving Efficiency of SVM k-fold Cross-validation by Alpha Seeding.

2. He K, Zhang X, Ren S, Sun J. Deep residual learning for image recognition. 770-8.

Supplementary Figure 1: The qualified images of 26 anatomical landmarks of the upper digestive tract.


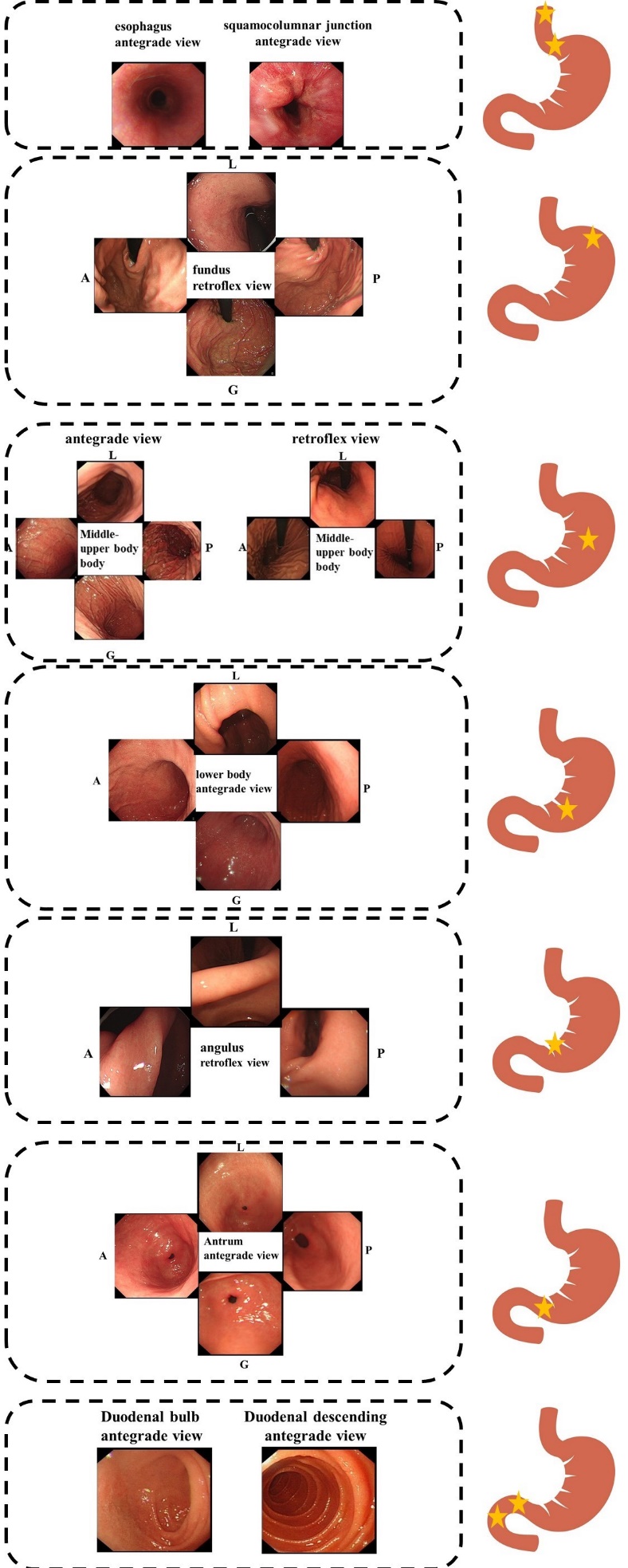


Supplementary Figure 2: the confusion matrix of the CNN.


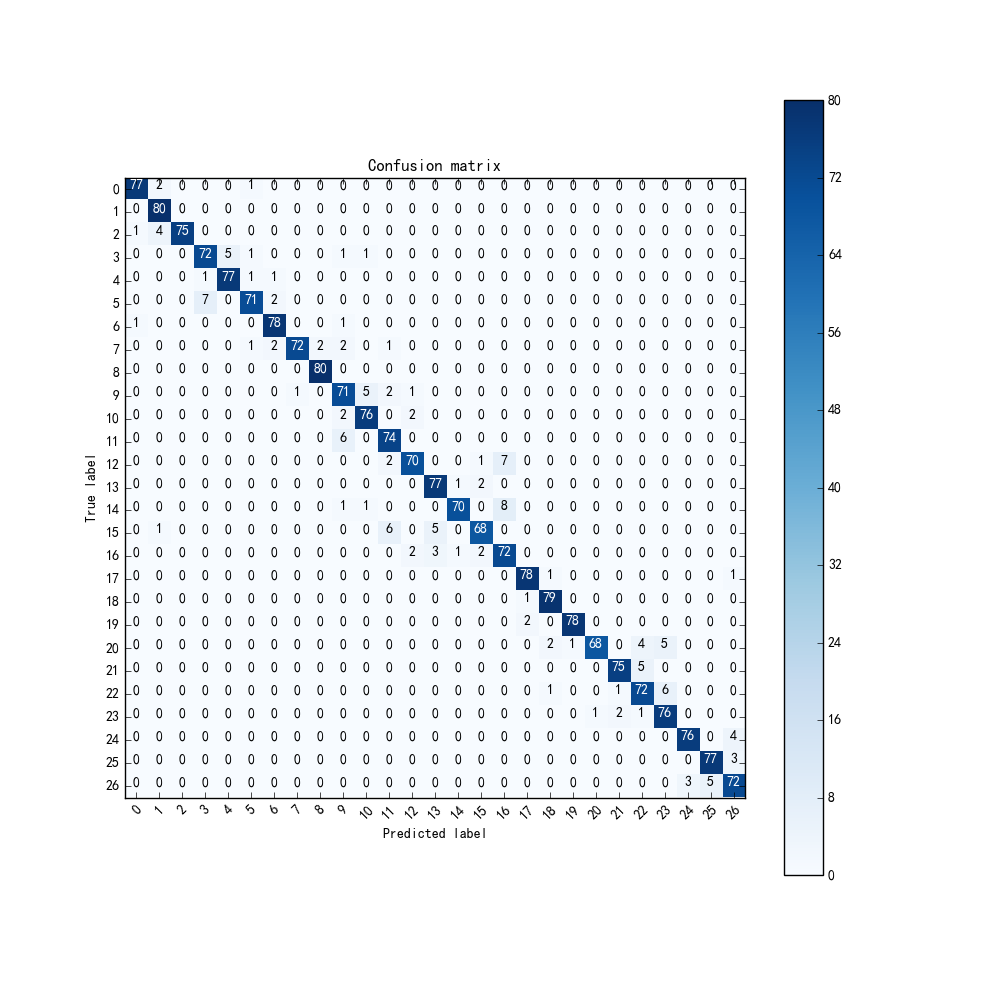


Supplementary figure 3: The flowchart of patient recruitment.


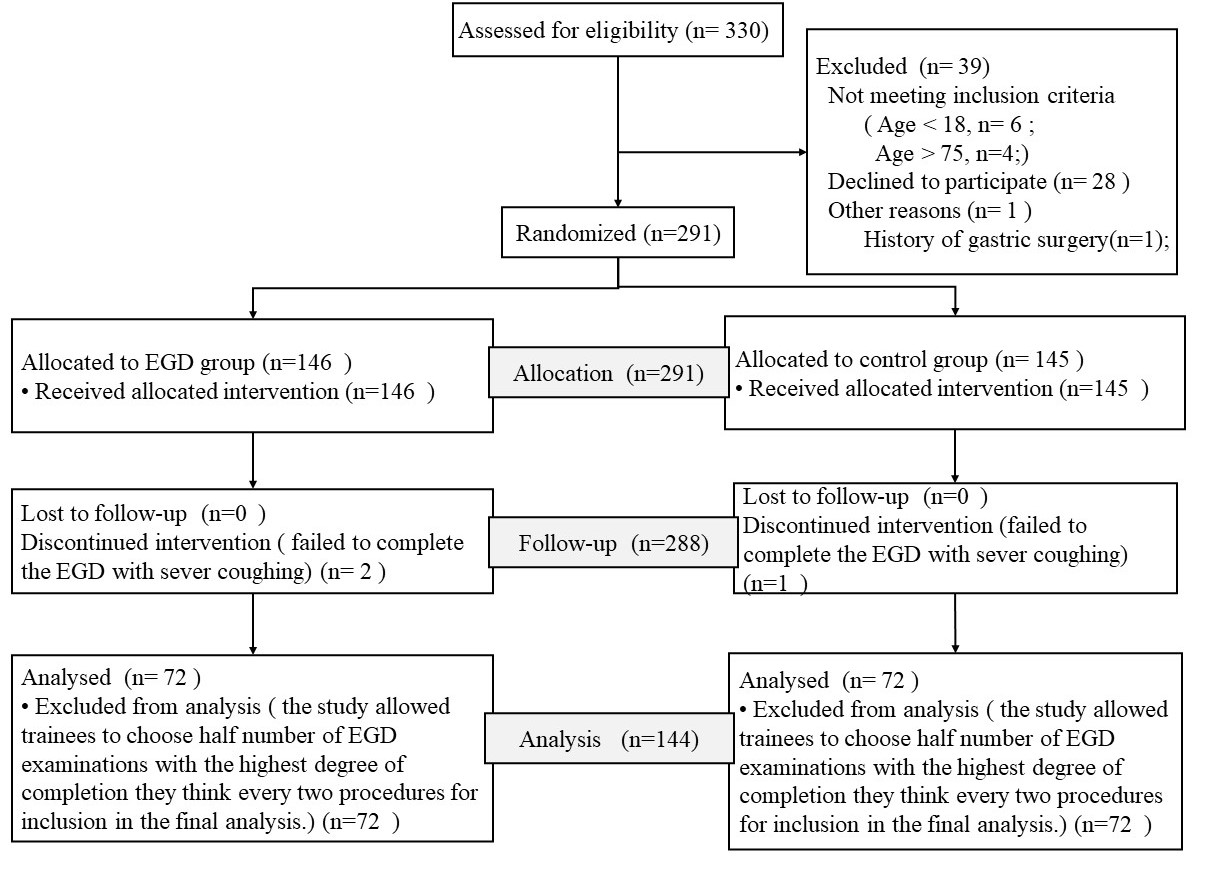


Supplementary Table 1: The sample distribution in each classification of anatomical landmarks of the upper digestive tract.

| **Sites** | **Number (training datasets)** | **Accuary** |
| --- | --- | --- |
| 0-NA | 2632 | 00 acc: 96.2% |
| 1-esophagus | 3063 | 01 acc: 100.0% |
| 2-squamocolumnar junction | 2831 | 02 acc: 93.8% |
| 3-antrum (G) | 3204 | 03 acc: 90.0% |
| 4-antrum (P) | 3109 | 04 acc: 96.2% |
| 5-antrum (A) | 3551 | 05 acc: 88.8% |
| 6-antrum (L) | 3484 | 06 acc: 97.5% |
| 7-duodenal bulb | 2502 | 07 acc: 90.0% |
| 8-duodenal descending | 2720 | 08 acc: 100.0% |
| 9-lower body (G) | 2477 | 09 acc: 88.8% |
| 10-lower body (P) | 2390 | 10 acc: 95.0% |
| 11-lower body (A) | 2291 | 11 acc: 92.5% |
| 12-lower body (L) | 2300 | 12 acc: 87.5% |
| 13-middle-upper body (F) (G) | 3847 | 13 acc: 96.2% |
| 14-middle-upper body (F) (P) | 2139 | 14 acc: 87.5% |
| 15-middle-upper body (F) (A) | 2040 | 15 acc: 85.0% |
| 16-middle-upper body (F) (L) | 3199 | 16 acc: 90.0% |
| 17-fundus (G) | 3173 | 17 acc: 97.5% |
| 18-fundus (P) | 2419 | 18 acc: 98.8% |
| 19- fundus (A) | 3942 | 19 acc: 97.5% |
| 20-fundus (L) | 2151 | 20 acc: 85.0% |
| 21-middle-upper body (R) (P) | 2067 | 21 acc: 93.8% |
| 22-middle-upper body (R) (A) | 2322 | 22 acc: 90.0% |
| 23-middle-upper body (R) (L) | 3043 | 23 acc: 95.0% |
| 24-incisura (P) | 3247 | 24 acc: 95.0% |
| 25-incisura (A) | 3055 | 25 acc: 96.2% |
| 26-incisura (L) | 2544 | 26 acc: 90.0% |
| total | 75742 | overall acc: 93.1% |
